# An antibody response to human polyomavirus 15-mer peptides is highly abundant in healthy human subjects

**DOI:** 10.1186/1743-422X-10-192

**Published:** 2013-06-12

**Authors:** Lieven J Stuyver, Tobias Verbeke, Tom Van Loy, Ellen Van Gulck, Luc Tritsmans

**Affiliations:** 1 Janssen Diagnostics, Turnhoutsebaan 30, Beerse, B-2340, Belgium; 2 Open Analytics, Antwerpen, Belgium; 3 Janssen Infectious Disease –CREATe, Beerse, Belgium; 4 Janssen R&D, Beerse, Belgium

**Keywords:** Human polyomaviruses, Peptide microarray, Pentapeptides epitopes

## Abstract

**Background:**

Human polyomaviruses (HPyV) infections cause mostly unapparent or mild primary infections, followed by lifelong nonpathogenic persistence. HPyV, and specifically JCPyV, are known to co-diverge with their host, implying a slow rate of viral evolution and a large timescale of virus/host co-existence. Recent bio-informatic reports showed a large level of peptide homology between JCPyV and the human proteome. In this study, the antibody response to PyV peptides is evaluated.

**Methods:**

The in-silico analysis of the HPyV proteome was followed by peptide microarray serology. A HPyV-peptide microarray containing 4,284 peptides was designed and covered 10 polyomavirus proteomes. Plasma samples from 49 healthy subjects were tested against these peptides.

**Results:**

In-silico analysis of all possible HPyV 5-mer amino acid sequences were compared to the human proteome, and 1,609 unique motifs are presented. Assuming a linear epitope being as small as a pentapeptide, on average 9.3% of the polyomavirus proteome is unique and could be recognized by the host as non-self. Small t Ag (stAg) contains a significantly higher percentage of unique pentapeptides. Experimental evidence for the presence of antibodies against HPyV 15-mer peptides in healthy subjects resulted in the following observations: i) antibody responses against stAg were significantly elevated, and against viral protein 2 (VP2) significantly reduced; and ii) there was a significant correlation between the increasing number of embedded unique HPyV penta-peptides and the increase in microarray fluorescent signal.

**Conclusion:**

The anti-peptide HPyV-antibodies in healthy subjects are preferably directed against the penta-peptide derived unique fraction of the viral proteome.

## Background

The *Polyomaviridae* are a family of non-enveloped circular double-stranded DNA viruses. The Polyomaviridae Study Group of the International Committee on Taxonomy of Viruses (ICTV) has proposed that the Polyomaviridae family will be comprised of three genera: two genera containing mammalian viruses (Orthopolyomavirus and Wukipolyomavirus) and one genus containing avian viruses (Avipolyomavirus) [1]. Besides the HPyVs that were discovered more than 40 years ago (JCPyV and BKPyV), several new polyomaviruses have been discovered over the last 7 years in human clinical samples, namely WUPyV [2], KIPyV [3], MCPyV [4], TSPyV [5], HPyV6 and HPyV7 [6], HPyV9 [7], HPyV10 [8] and MWPyV [9], STLPyV [10], and HPyV12 [11]. Based on pairwise percentage identity of the viral protein-1 (VP1) open reading frame, members of the same species have more than 90% identity, between species identity ranged from 61 to 85%, and viruses belonging to different genera have less than 61% identity [6]. The primate virus SV40 has been detected in human samples [12], but there is inadequate evidence about the relationship to human carcinogenesis [13]. The recently discovered human virus (HPyV9) is closely related to the African Green Monkey Lymphotropic PyV (LPyV) [7,14], and this discovery might explain the previously observed serological evidence that LPyV-like virus infections may occur in humans [15,16].

Multiple methods have been used to measure antibodies to polyomavirus virions. The most common method is based on the use of baculovirus-expressed VP1 virus-like-particles (VLP) in an enzyme immuno assay (EIA) [17-20]. Additionally, there are E.coli-expressed VP1 proteins that do not form VLP, but rather pentameric VP1 capsomers either used in an EIA, or in a Luminex multiplex platform [15,21]. Currently, the STRATIFY JCPyV ELISA is the only Food and Drug Administration (FDA) approved assay for JCPyV [22], while all the others are lab developed tests for ‘research use only’. To a large extent, the immune response measured in these VLP-, or capsomer-based assays is directed against conformational epitopes [23]. There are few peptide EIA described that are presumably detecting linear epitopes/mimitopes [12].

Since there is considerable homology at the VP1 region for the human PyV belonging to the same genus, it does not come as a surprise that there is a considerable cross-reactivity in serological assays [23]. For example, serological cross-reactivity in the alpha-PyV is explained by 77% amino acid identity between JCPyV and SV40, 83% between BKPyV and SV40, and 80% between JCPyV and BKPyV. The availability of VLP of the different PyV allows to conduct inhibition studies, and find virus specific-antibodies [16,23].

By using phylogenetic methods, the worldwide distribution of JCPyV genotypes was found to mirror the migrations and genetics of the human family [24,25]. JCPyV, and most likely many other polyomaviruses, have co-evolved with their hosts over long evolutionary timescale, which allowed mechanisms of immune-evasion to be evolved. Indeed, analysis of JCPyV polyprotein for peptide sharing with the human proteome revealed that the virus has hundreds of pentapeptides sequences in common with the human proteins [26]. This type of immune-evasion may contribute to the asymptomatic character of the primary infection, and subsequent latency. But, several sequence domains that are JCPyV-unique were also detected [26]. The role of these unique domains in the mechanisms and molecular basis for polyomavirus reactivation and pathogenesis remains unclear.

Since there is a great overlap in pentapeptide sequences between the human genome and the PyV genome, it is of particular interest to distinguish between domains that are recognized by auto-antibodies, and other domains that are characteristic for a polyomavirus infection. Therefore, in this study, we explored the following items: i) is there an immune response to HPyV-epitopes presented as peptides; ii) how do these peptide epitopes relate to unique viral domains with no overlap with the human proteome. The answers to these questions could help in understanding the immune response to HPyV infections, the discrimination between ‘self’ and ‘non-self’, the status of an uninfected individual, and hopefully contribute to the unraveling of the mechanisms underlying virus reactivation.

## Results

### Polyomavirus peptide similarity with the human proteome

The HPyV reference sequence database was retrieved from NCBI. The viral proteins LTAg, stAg, VP1, and VP2 for 11 HPyV were cut *in silico* into either 5-mer (with 4 amino acid (aa) overlap), 6-mer (with 5 aa overlap), or 7-mer (with 6 aa overlap) peptides. This resulted into 17,396 penta-peptides, 17,347 hexa-peptides, and 17,304 hepta-peptides. These small peptides were presented to the complete human proteome (20,227 proteins in http://www.uniprot.org/faq/48) for pairwise comparison (in order to identify correct matches).

**Figure 1:**
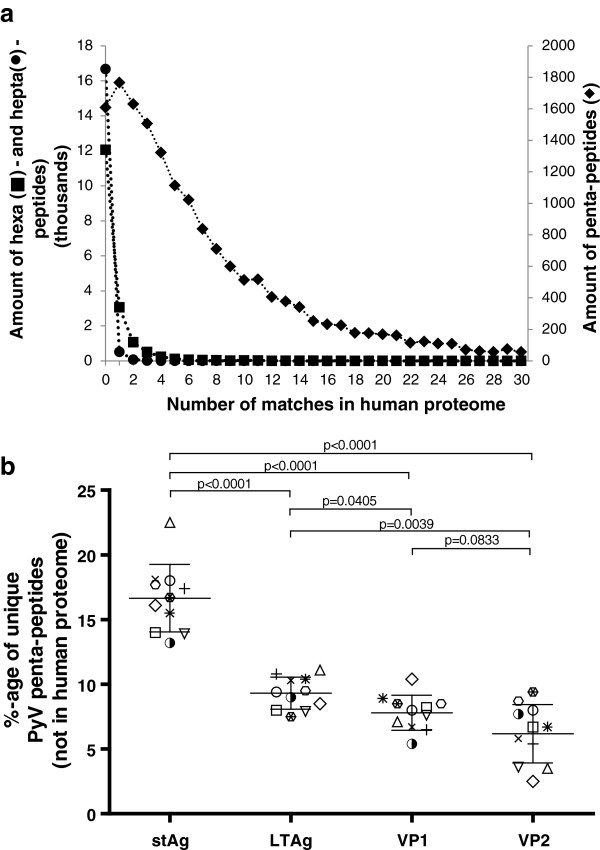
**In silico analysis of the HPyV unique peptide sequences as compared to the human proteome. a**. The polyomavirus proteome was presented as 17,396 pentapeptides, 17,347 hexapeptides, and 17,304 heptapeptides to the complete human proteome. The number of polyomavirus peptides with zero up to 30 matches in the human genome are given, with values for hexa- and hepta-peptides numbers on the primary Y-axis (left), while values for pentapeptides are shown on the secondary Y-axis (right). **b**. Percentage of HPyV penta-peptides with no match in the human proteome. The number of “zero hit” pentapeptides was divided by the total number of amino acids for each viral protein (protein length differ between viral species). Each data-cluster contains 11 data-points, corresponding to the following viruses: JCV (□, n = 134 unique pentapeptides), BKV (○, n = 155), SV40 (n = 150), KIV (△, n = 155), WUV (▽, n = 121), MCV (&z.diam;, n = 147), MWV (✖, n = 150), TSV (n = 160), HPyV6 (✚, n = 148), HPyV7 (❋, n = 156), and HPyV9 (◗, n = 133). The means (16.6%; 9.3%; 7.8%; and 6.2%; respectively) with standard deviations are shown.

A total of 1,609 (9.25%) penta-peptides had no match in the human genome, while for the hexa- and hepta-peptides, the numbers rose to 12,064 (69.5%), and 16,679 (96.39%), respectively. The distribution expressed in number of matches of hexa- and hepta-peptides follows a similar pattern, but a very different one as compared to the pentapeptides (Figure 1a). The degree of uniqueness and the sharp drop with increasing number of matches on the human genome suggest that hexa- and hepta-peptides are likely to be HPyV-specific. Consequently, if an epitope would encompass 6 or more amino acids in one continuous stretch, this epitope is also likely to be HPyV-specific. However, for the penta-peptides, the distribution is rather different, as only 9.25% of the peptides were found to be unique to polyomaviruses. The remaining 90.75% of peptides have at least one or more matches with the human proteome. There were 939 penta-peptides, 11 hexa-peptides, and 2 hepta-peptides with more than 30 matches in the human proteome (not shown); these motifs were often stretches containing 3 to 6 identical amino acids, like for example hexapeptide AAAAAA in the HPyV7 VP1 carboxyterminal region (… ^356^SSNAAAAAAKISVA^370^P…), which was found 2,364 times in the human proteome.

The 1,609 penta-peptides with no match in the human proteome were distributed as follows: JCPyV: n = 134, BKPyV: n = 155, SV40: n = 150, KIPyV: n = 155, WUPyV: n = 121, MCPyV: n = 147, MWPyV: n = 150, TSPyV: n = 160, HPyV6: n = 148, HPyV7: n = 156, and HPyV9: n = 133. When ranked per protein, there were 700, 343, 326, 231, and 9 pentapeptides for LTAg, stAg, VP1, VP2, and agno, respectively with no match in the human proteome (Additional file 1). From the above data, one can calculate the numbers of peptides with “no match” in the human proteome, and this is expressed as ‘percentage of unique polyomavirus penta-peptides per virus per protein (Figure 1b). This figure shows that the mean of 16.6% for all PyV in the case of stAg is significantly higher (p < 0.0001) than the 9.3%; 7.8%; and 6.2%; respectively for LTAg, VP1, and VP2. Differences between the other means, except VP1 *versus* VP2, were also found to be significantly different from each other (p < 0.05).

### Array results

**Figure 2:**
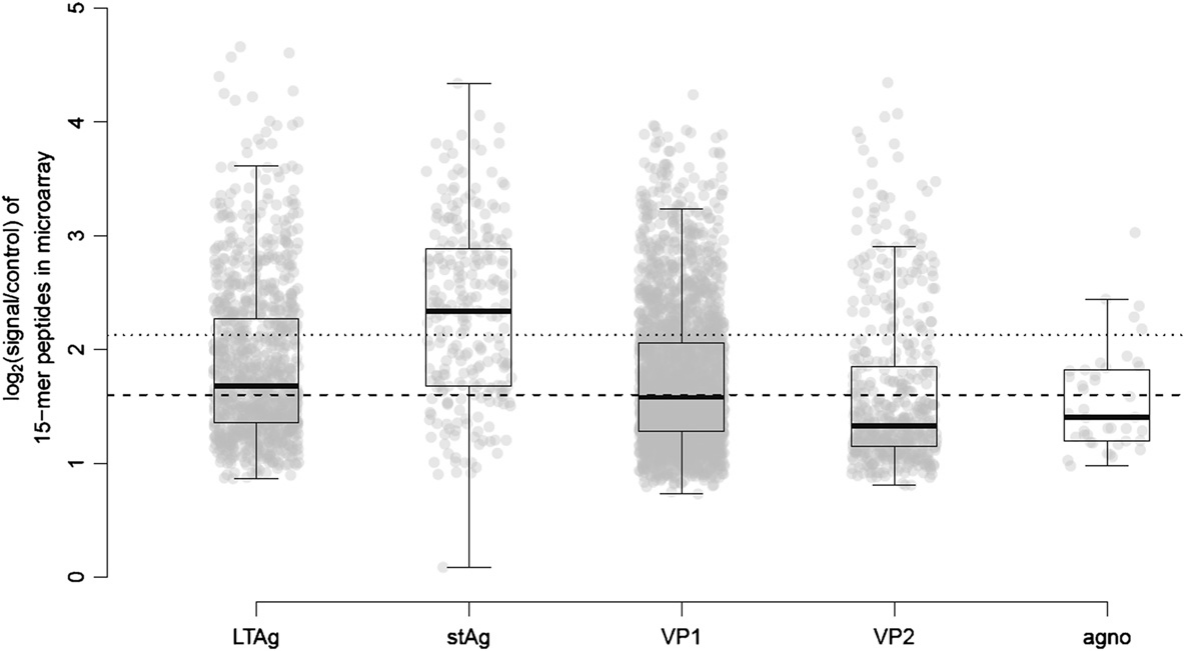
**Distribution of the polyomavirus peptide microarray signals in function of the the viral proteins.** Values on the Y-axis are expressed as log_2_(signal of sample/signal of the no-sample control), making a value of 0 = background. From the statistics for all 4284 datapoints: median: 1.593 (dashed line); 75^th^ percentile: 2.129 (dotted line). The following median values, respectively from left (LTAg) to right (agno), were found: 1.68, 2.33, 1.58, 1.33, and 1.41. The median value from one protein was compared to the median value all other proteins by using a “Linear Rank test for two samples”. The estimated difference between the medians (= median test protein *minus* median all other datapoints), the lower (LCI) and upper (UCI) 95% confidence intervals, and the p-values are as follows: 1) LTAg – others: 0.10, LCI: 0.032, UCI: 0.157, p = 0.0006; 2) stAg – others: 0.76, LCI: 0.587, UCI: 0.866, **p = 3.45E-23**; 3) VP1 – others: -0.05, LCI: -0.108, UCI: -0.005, p = 0.02712; 4) VP2 – others: -0.31, LCI: -0.360, UCI: -0.260, **p = 4.44E-16**; and 5) agno – others: -0.20, LCI: -0.323, UCI: 0.129, p = 0.2727.

A total of 4,284 peptides were incubated in a peptide microarray format with plasma samples from 49 HVs, resulting in 209,916 data points. This population has a median log_2_ (signal/control) value of 1.683 (min: -1.222, 25^th^: 1.135; 75^th^: 2.50; 90^th^: 3.319; and max: 6.909). We used the median values of 49 HV data points for each peptide to generate the figures used in this article. On this population of 4284 data points, the following values were obtained: minimum: 0.089; 25^th^ percentile: 1.281; median: 1.593; 75^th^ percentile: 2.129; maximum: 4.659; mean: 1.778; standard deviation: 0.663; standard error: 0.010; the lower 95% confidence interval (CI) of mean: 1.758; and the upper 95% CI of mean: 1.798. The distribution of the signal intensity for the 10 different viruses did not result in any specific observation (data not shown). But when analyzing the 5 different proteins (LTAg, stAg, VP1, VP2, and agnoprotein) that were present as peptides on the microarray, it was surprising to see that antibody responses to stAg peptide were significantly elevated (p = 3.45E-23), but also that the anti-peptide antibody responses for VP2 were significantly less abundant (p = 4.44E-16) (Figure 2).

### Correlation between ‘unique polyomavirus peptides (not present in the human proteome)’ and ‘peptide microarray results’

Linear peptide epitopes are most frequently between 7 – 9 amino acids long (range 4 – 12) [27]. We focused on 5- to 7-mer peptides. Figure 1 illustrated already that most of the hexa- and hepta-peptides are virus-specific, and in these cases, linear epitopes would likely be virus-specific. The analysis of the 5-mer peptides is less virus-restricted. Since the microarray peptides were 15-mers, this means that up to 11 5-mer epitopes could be present on one single peptide. Some of these 11 epitopes might be virus-specific, but others might have identical motifs in the human genome.

**Figure 3:**
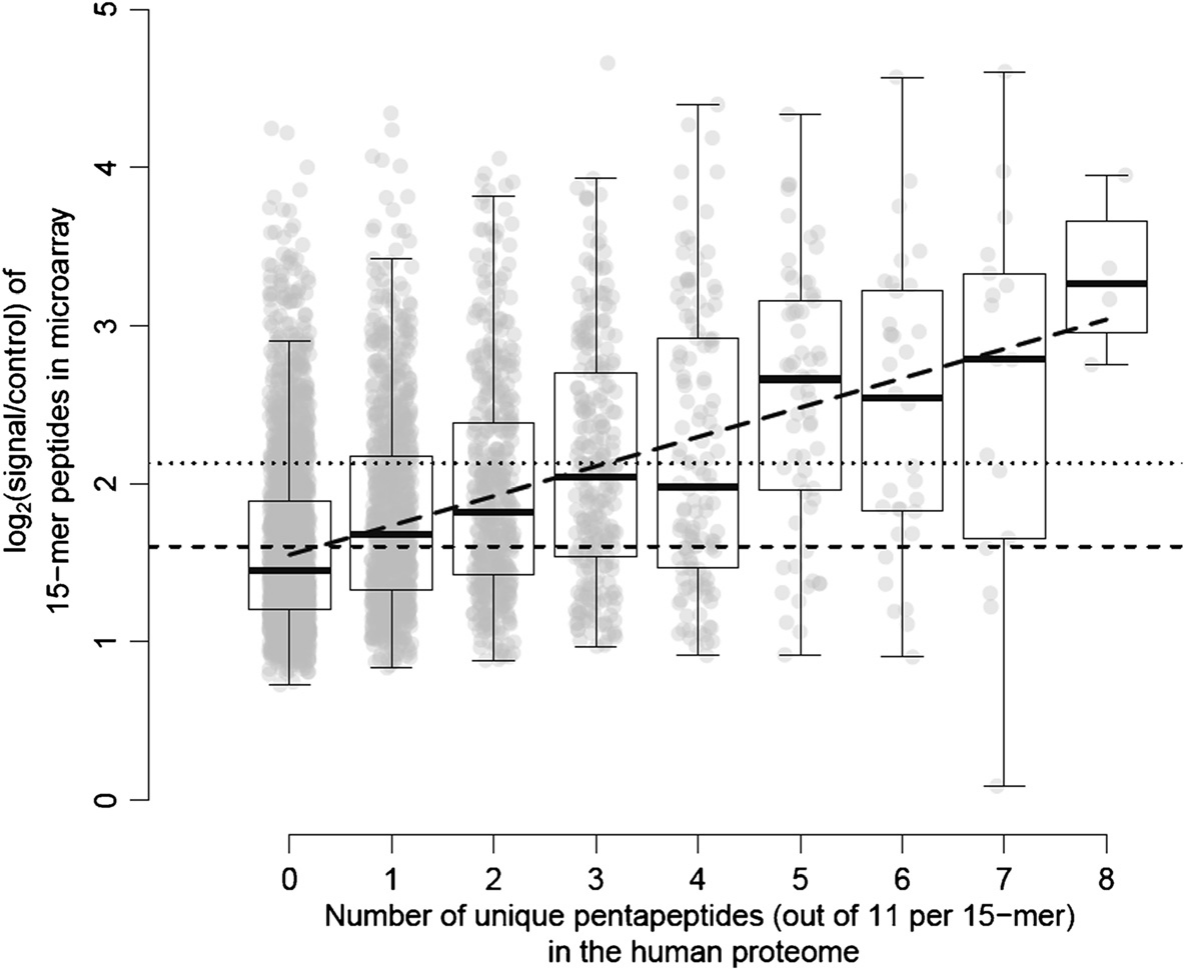
**Correlation plot between the median HS microarray signals in function of the amount of pentapeptides with no match in the human proteome.** There were in total 2276, 980, 495, 269, 136, 70, 36, 18, and 4 ‘15-mer’ peptides with, respectively, 0, 1, 2, 3, 4, 5, 6, 7, and 8 5-mer peptide “no matches” in the human proteome. The dotted/dashed lines represent, respectively, the 75th percentile at 2.129, and the median at 1.593 of the complete population. The boxplot median values are, respectively, 1.449, 1.681, 1.820, 2.043, 1,979, 2.661, 2.539, 2.788, and 3.265. All data-points were used in a robust linear regression analysis tool to calculate the slope (0.17), Y intercept (1.55) and the lower (0.14) and upper (0.18) confidence intervals on the slope. The linear regression line is drawn as in interrupted line through the boxplots.

Therefore, results from the 4,284 peptides on the microarray were interpreted as a summary signal of 11 5-mer peptides, under the assumption that microarray peptides with viral-specific 5-mer epitopes would result in higher signals. As can be deduced from Figure 3, there was indeed a correlation between the ‘number of embedded penta-peptides with no human homologue’ in the microarray peptides and the strength of the signal obtained with human HS plasma. Based on the linear regression analysis using all data-points, there was a stepwise increase in expression value as given by the following formula: y = 0.17× + 1.55 (Table 1). The difference between each subset is significant (p < 0.05). The 95% CI on the slope were within 0.14 and 0.18. In addition, Table 1 provides the slopes and intercepts for each protein and virus separated. When ranking the groups according to the steepest slope, KIPyV and JCPyV were seen as the 2 most important contributors to the overall slope. Despite the fact that agno had only a small amount of unique peptides, the slope turned out to be very steep. In contrast, the slope was rather shallow for BKPyV and SV40, and stAg. The Y-intercept was highest for stAg (2.09, in agreement with the observation in Figure 2). In conclusion, microarray peptides with one or more embedded polyomavirus penta-peptides with no human homologue showed a higher signal on the microarray, and therefore are likely to represent viral-specific epitopes.

**Table 1:** Linear regression data on correlation plots

	**Penta-peptides**			
	**Slope**	**Intercept**	**95% CI on slope**	
**Virus/protein**			**Lower**	**Upper**
all	0.17	1.55	0.14	0.18
KIV	0.27	1.46	0.23	0.31
JCV	0.20	1.56	0.15	0.24
HPyV7	0.16	1.57	0.03	0.29
MCV	0.16	1.49	0.10	0.21
TSV	0.15	1.88	0.03	0.28
WUV	0.15	1.49	0.06	0.24
HPyV9	0.14	1.92	-0.04	0.31
HPyV6	0.14	1.73	0.01	0.26
BKV	0.12	1.50	0.09	0.16
SV40	0.12	1.63	0.06	0.18
agno	0.24	1.38	0.04	0.44
VP2	0.16	1.28	0.09	0.22
LTAg	0.16	1.64	0.10	0.21
VP1	0.13	1.54	0.11	0.16
stAg	0.12	2.09	0.08	0.17

Linear regression data on correlation plots between median HS microarray signals in function of the amount of pentapeptides with no match in the human proteome. Values in this table were obtained by applying a robust linear model based on the MM-type regression estimator (to control the influence of outliers) [28,29].

## Discussion

The results of the human proteome scan can be summarized as follows; i) if 5-mer peptides are considered, up to 90.75% of the viral proteome is similar to the human proteome, and therefore seen as “self”, but the percentage of ‘self’ drops to 30.5% with 6-mer peptides, and to 3.61% with 7-mer peptides [26]; and ii) with an average of 16.6% of unique pentapeptides, stAg is significantly less recognized as ‘self’ as compared to the other viral proteins; while VP2 proteins showed with 6.2% the highest degree of homology with the host.

The functionalities of stAg have been reviewed previously [30]. The evidence collected for stAg in this paper showed some specific features for this viral protein, suggesting that the protein has not evolved towards a higher percentage of “host self” (Figure 1b, 16.6% of unique pentapeptides), and thereby maintaining an elevated level of immune presentation and antibody generation against linear epitopes (Figure 2). This might be advantageous for diagnostic purposes, but does not educate on the pathological consequences. Opposite to stAg is the observation for VP2, for which it seems like there is an evolution towards an ‘as high as possible’ “self” (host) content, thereby reducing the immune response. This is unexpected, because VP2, as minor part of the - in majority VP1 composed - viral structure, must be one of the first proteins that are recognized by the immune system upon infection or exposure. A potential explanation might be that VP2 is crucial in structure and function, and therefore has to evolve towards a protein that is not or poorly immune-dominant (a pressure that is not or less evident for stAg). Note that these stAg and VP2 considerations were based on median values obtained on peptide microarrays.

In a previous study [26], pentamer domains were suggested to be desired motifs for eventual vaccine development. Our results however suggest that there is already a significant amount of antibodies build against these motifs in healthy volunteers, and thus it seems like a redundant approach to target unique pentamer motifs. Figure 3 also shows that there is a large fraction of peptides without unique pentapeptides that nevertheless showed high median signal intensity. This can be explained by either the fact that it does not need to be unique to be an epitope, or that the reactivity is against embedded linear epitopes that are 6-mer, 7-mers, or longer. An antibody response against a non-unique domain would be seen as an auto-immune response. The recent development of antigen microarray chip technology for detecting global patterns of antibody reactivities makes it possible to study the natural autoimmune repertories within healthy humans, the so called ‘immunological homunculus (immunculus)’ [31]. The immunculus is considered as the general network of constitutively expressed natural auto-antibodies against extracellular, membrane, cytoplasmic, and nuclear self-antigens (ubiquitous and organ-specific). The repertoires of natural auto-antibodies are surprisingly constant in healthy persons, independent of gender and age, and characterized by only minimal individual peculiarities [31,32]. Our approach however does not allow concluding whether the signals were against larger epitopes, or against ‘self’ domains and be part of the “immunculus”. Therefore, we cannot exclude that auto-antibodies for peptide motifs encoded in the human proteome are responsible for the cross-reactivity (immunological homunculus), or that some of the microarray signals could be explained by non-specific binding (see below).

Previously, it was shown that the immune reactivity of human sera directed against native VP1 is far more important as compared to the denatured form of VP1 [33,34]. The fact that peptide microarrays sometimes gave high signals (Figures 2 and 3) is therefore at variance with the observations made in the literature. The biological meaning of the presence of antibodies against linear HPyV epitopes is unclear. One hypothesis might be that, besides the viral particle that presents conformational epitopes to the immune system, there is quite some presentation of degraded viral protein in form of small peptides, and in case this is a unique motif (= unique pentapeptide), the immune system is building a detectable immune response. It is of particular importance to note here that we could illustrate the presence of antibodies against linear epitopes – mainly against viral unique pentapeptide fraction – against not only VP1, but also LTAg, stAg, and VP2, proteins that are only present as a consequence of a replication cycle (and not merely exposure).

Obviously the large number of peptides on the microarray makes it impractical and technologically almost impossible to be evaluated and/or confirmed in ELISA. Some confirmatory examples will be published elsewhere. In our opinion, the only way for future validation of all these possible epitope regions is by careful selection of significantly contributing peptides, and testing them on validated peptide microarray platforms. Despite the research progress that has been made by using peptide microarrays [27,35-37], there is still hesitation to use these arrays beyond the initial screening, because of possibilities of a-specific reactivities, lack of reliable relation between signal intensities and antibody affinities, lack of array production reproducibility, and intra- and inter-assay variability. In order to evaluate larger panels of donors, patients, and certain risk groups against a large panel of HPyV peptides, array optimization will be required. Despite this, several other groups have tried to use peptide microarrays to miniaturize the antigen-antibody interaction while simultaneously studying several peptide sequences, e.g. in the field of GB virus C, Herpes simplex, and human coronaviruses. They concluded that antigenic peptides could be considered useful tools for designing new diagnostic systems with often sensitivities in the range of low- picomolar concentrations of mAbs and with a high specificity [38,39]. However, while evaluating our results, we were absolutely aware of the shortcomings of the initial experiments. But because the presentation of our results was population-based, and mainly derived from the median values, the observed tendencies were considered reliable, and will be used for future work and confirmations.

## Conclusion

In this study, in essence 2 different topics were evaluated, namely: the correlation between the polyomavirus proteome in relation to the human proteome, and the study of a HPyV peptide microarray incubated with human plasma samples obtained from healthy subjects. A correlation between the presence of unique pentapeptides motifs embedded in 15-mer peptides and the signal obtained on the microarray was presented. Under the assumption that a linear epitope could be as small as a pentapeptide, on average 9.3% of the polyomavirus proteome is unique and could be recognized by the host as non-self and it is specifically against these 9.3% of unique motifs that the immune response has been seen.

## Methods and materials

### Healthy subject (HS) samples

A total of 49 healthy subjects were included in this study. For this study, the protocol and the informed consent document has been submitted to the “Commissie voor Medische Ethiek – ZiekenhuisNetwork Antwerpen (ZNA)” and approved (E.C. Approval No 3792). Informed consent was available for all 49. There were 28 women (age between 23 and 54 years, with mean ± SEM = 39.8 ± 1.8), and 21 men (age between 27 and 57, with mean ± SEM 42.6 ± 2.0). HS were selected to represent different geographical areas: 20 HS were born in Belgium, 3 in Romania and 2 in India, 2 from – respectively - South-Africa, the UK, and USA, and 1 from each of the following countries: Burundi, China, Democratic Republic of Congo, Egypt, Germany, Hong Kong, Israel, Italy, Jamaica, Japan, Kenya, Macedonia, Morocco, Slovakia, South Korea, Sri Lanka, The Netherlands, and Ukraine. Each HS gave 50 ml of blood, and plasma was divided in 300 μl aliquots and stored at -80°C.

### Polyomavirus peptide microarrays

A peptide array representing the human polyomavirus proteome was prepared by JPT Innovative Peptide Solutions (Berlin, Germany), as well as all experiments and data collection. Polyomavirus protein sequences were retrieved from the NCBI database. The 6 best covering sequences for each protein of each virus was calculated. The following proteins were included: agnoprotein (agno), small T antigen (stAg), large T antigen (LTAg), VP1, VP2, VP3 of the viruses BKPyV, JCPyV, KIPyV, WUPyV, MCPyV, and SV40. In addition, the VP1 protein of the viruses HPyV6, HPyV7, HPyV9, and TSPyV were also presented. For JCPyV VP1, a 100% coverage of published variants were covered by peptides, while for all other proteins, a coverage of at least 95% was obtained. This resulted in an array of 4,284 15-mer peptides, overlapping by 11 residues. Each peptide was displayed in triplicates on one single array chip (3 sub-arrays). The peptide microarray was incubated with a primary antibody or subject serum, followed by incubation with a fluorescently labeled secondary antibody. Read-out was done by scanning the array by means of a fluorescent microscope. Several control incubations (no primary antibody) and control spots (human IgG) were included. The full procedure of the assay was as described by the microarray provider (JPT, Berlin, Germany). The triplicate quantitative values for each peptide were averaged, and one single value used for further analysis. All imaging and data manipulation was performed as described by JPT Innovative Peptide Solutions (Berlin, Germany). The data presented in this manuscript are log_2_(test peptide/control) values, derived from the original fluorescent values. Mapping (annotations) of the peptides was done against reference NCBI database sequences: JCPyV MAD1 (AAA82102, AAA82101, AAA82099, and AAA82103 for LTAg, VP1, VP2, and stAg, respectively), BKPyV Dunlop (CAA24300, CAA24299, CAA24297, and CAA24301), SV40 (YP_003708382, YP_003708381, YP_003708379, and YP_003708383), KIPyV CU-258 (ACB12028, ACB12026, ACB12024, and ACB12027), WUPyV CU-302 (ACB12038, ACB12036, ACB12034, and ACB12037), and MCPyV HF (AEM01097, AEM01098, AEM01099, AEM01096).

### Bio-informatic analysis

Each of the 4284 15-mer sequences on the polyoma peptide JPT array were scanned for hits against the Uniprot human complete proteome [ http://www.uniprot.org/uniprot/?query=organism%3a9606+AND+keyword%3a%22Complete+proteome+%5bKW-0181%5d%22+reviewed%3ayes&force=yes&format=fasta and motivated by http://www.uniprot.org/faq/48] using R [40] and BioConductor [41,42]. Every 15-mer was scanned against every human protein and only exact matches were taken into account to compute the number of hits. The peptide array data were annotated with these hits and the joint information was used for all subsequent analyses. For all analyses involving micro-array intensities, the values were expressed as log2 (sample/control). Despite the transformation, the data still displayed slight skew (to the right). In order to take this into account, methods that are robust against such skew as well as outlying values have been used throughout the analysis. Descriptive statistics were performed using base R [40]. Comparisons of medians made use of linear rank methods as made available in [43]. The assessment of the relationship between presence in the human genome and signal intensity on the microarrays made use of robust linear models with MM-type estimators as implemented in Rousseeuw et al., 2011 [44].

## Competing interest

The authors declare that they have no competing interests.

## Authors’ contributions

LJS and LT are responsible for the design of the study, supervising the activities, and writing of the manuscript. TV carried out the in silico analysis, bioinformatic work and statistical analysis; TVL and EVG participated in the experimental approaches. All authors read and approved the final manuscript.

## Supplementary Material

Additional file 1 **Unique polyomavirus pentapeptides sequences.** List of polyomavirus-derived pentapeptides with no homology to the human proteome. Click here for file

## References

[B1] Johne R Buck CB Allander T Atwood WJ Garcea RL Imperiale MJ Major EO Ramqvist T Norkin LC Taxonomical developments in the family Polyomaviridae Arch Virol 2012 156 1627 1634 21562881 10.1007/s00705-011-1008-x PMC3815707

[B2] Gaynor AM Nissen MD Whiley DM Mackay IM Lambert SB Wu G Brennan DC Storch GA Sloots TP Wang D Identification of a novel polyomavirus from patients with acute respiratory tract infections PLoS Pathog 2007 3 e64 10.1371/journal.ppat.0030064 17480120 PMC1864993

[B3] Allander T Andreasson K Gupta S Bjerkner A Bogdanovic G Persson MA Dalianis T Ramqvist T Andersson B Identification of a third human polyomavirus J Virol 2007 81 4130 4136 10.1128/JVI.00028-07 17287263 PMC1866148

[B4] Feng H Shuda M Chang Y Moore PS Clonal integration of a polyomavirus in human Merkel cell carcinoma Science 2008 319 1096 1100 10.1126/science.1152586 18202256 PMC2740911

[B5] van der Meijden E Janssens RW Lauber C Bouwes Bavinck JN Gorbalenya AE Feltkamp MC Discovery of a new human polyomavirus associated with trichodysplasia spinulosa in an immunocompromized patient PLoS Pathog 2010 6 e1001024 10.1371/journal.ppat.1001024 20686659 PMC2912394

[B6] Schowalter RM Pastrana DV Pumphrey KA Moyer AL Buck CB Merkel cell polyomavirus and two previously unknown polyomaviruses are chronically shed from human skin Cell Host Microbe 2010 7 509 515 10.1016/j.chom.2010.05.006 20542254 PMC2919322

[B7] Scuda N Hofmann J Calvignac-Spencer S Ruprecht K Liman P Kuhn J Hengel H Ehlers B A novel human polyomavirus closely related to the african green monkey-derived lymphotropic polyomavirus J Virol 2011 85 4586 4590 10.1128/JVI.02602-10 21307194 PMC3126223

[B8] Buck CB Phan GQ Raiji MT Murphy PM McDermott DH McBride AA Complete genome sequence of a tenth human polyomavirus J Virol 2012 86 10887 10.1128/JVI.01690-12 22966183 PMC3457262

[B9] Siebrasse EA Reyes A Lim ES Zhao G Mkakosya RS Manary MJ Gordon JI Wang D Identification of MW Polyomavirus, a Novel Polyomavirus in Human Stool J Virol 2012 86 10321 10326 10.1128/JVI.01210-12 22740408 PMC3457274

[B10] Lim ES Reyes A Antonio M Saha D Ikumapayi UN Adeyemi M Stine OC Skelton R Brennan DC Mkakosya RS Discovery of STL polyomavirus, a polyomavirus of ancestral recombinant origin that encodes a unique T antigen by alternative splicing Virology 2013 436 295 303 10.1016/j.virol.2012.12.005 23276405 PMC3693558

[B11] Korup S Rietscher J Calvignac-Spencer S Trusch F Hofmann J Moens U Sauer I Voigt S Schmuck R Ehlers B Identification of a novel human polyomavirus in organs of the gastrointestinal tract PLoS One 2013 8 e58021 10.1371/journal.pone.0058021 23516426 PMC3596337

[B12] Corallini A Mazzoni E Taronna A Manfrini M Carandina G Guerra G Guaschino R Vaniglia F Magnani C Casali F Specific antibodies reacting with simian virus 40 capsid protein mimotopes in serum samples from healthy blood donors Hum Immunol 2012 73 502 510 10.1016/j.humimm.2012.02.009 22387152

[B13] Bouvard V Baan RA Grosse Y Lauby-Secretan B El Ghissassi F Benbrahim-Tallaa L Guha N Straif K Carcinogenicity of malaria and of some polyomaviruses Lancet Oncol 2012 13 339 340 10.1016/S1470-2045(12)70125-0 22577663

[B14] Sauvage V Foulongne V Cheval J Ar Gouilh M Pariente K Dereure O Manuguerra JC Richardson J Lecuit M Burguiere A Human polyomavirus related to African green monkey lymphotropic polyomavirus Emerg Infect Dis 2011 17 1364 1370 21801611 10.3201/eid1708.110278 PMC3381546

[B15] Kean JM Rao S Wang M Garcea RL Seroepidemiology of human polyomaviruses PLoS Pathog 2009 5 e1000363 10.1371/journal.ppat.1000363 19325891 PMC2655709

[B16] Viscidi RP Clayman B Serological cross reactivity between polyomavirus capsids Adv Exp Med Biol 2006 577 73 84 10.1007/0-387-32957-9_5 16626028

[B17] Plavina T Berman M Njenga M Crossman M Lerner M Gorelik L Simon K Schlain B Subramanyam M Multi-site analytical validation of an assay to detect anti-JCV antibodies in human serum and plasma J Clin Virol 2012 53 65 71 10.1016/j.jcv.2011.10.003 22104399

[B18] Rollison DE Helzlsouer KJ Lee JH Fulp W Clipp S Hoffman-Bolton JA Giuliano AR Platz EA Viscidi RP Prospective study of JC virus seroreactivity and the development of colorectal cancers and adenomas Cancer Epidemiol Biomarkers Prev 2009 18 1515 1523 10.1158/1055-9965.EPI-08-1119 19383887 PMC2743003

[B19] Bodaghi S Comoli P Bosch R Azzi A Gosert R Leuenberger D Ginevri F Hirsch HH Antibody responses to recombinant polyomavirus BK large T and VP1 proteins in young kidney transplant patients J Clin Microbiol 2009 47 2577 2585 10.1128/JCM.00030-09 19474265 PMC2725654

[B20] Hamilton RS Gravell M Major EO Comparison of antibody titers determined by hemagglutination inhibition and enzyme immunoassay for JC virus and BK virus J Clin Microbiol 2000 38 105 109 10618072 10.1128/jcm.38.1.105-109.2000 PMC86031

[B21] Antonsson A Green AC Mallitt KA O'Rourke PK Pawlita M Waterboer T Neale RE Prevalence and stability of antibodies to the BK and JC polyomaviruses: a long-term longitudinal study of Australians J Gen Virol 2010 91 1849 1853 10.1099/vir.0.020115-0 20219899

[B22] Food-and-Drug-Administration Anti- John Cunningham Virus (JCV) antibodies measured by Enzyme Linked Immunosorbent Assay (ELISA); 510(k) Number: K112394 http://www.accessdata.fda.gov/cdrh_docs/reviews/K112394.pdf 2012

[B23] Moens U Van Ghelue M Song X Ehlers B Serological cross-reactivity between human polyomaviruses Rev Med Virol 2013 10.1002/rmv.1747 23650080

[B24] Agostini HT Deckhut A Jobes DV Girones R Schlunck G Prost MG Frias C Perez-Trallero E Ryschkewitsch CF Stoner GL Genotypes of JC virus in East, Central and Southwest Europe J Gen Virol 2001 82 1221 1331 11297697 10.1099/0022-1317-82-5-1221

[B25] Sharp PM Simmonds P Evaluating the evidence for virus/host co-evolution Curr Opin Virol 2011 1 436 441 10.1016/j.coviro.2011.10.018 22440848

[B26] Lucchese G Confronting JC virus and Homo sapiens biological signatures Front Biosci 2013 18 716 724 10.2741/4133 23276955

[B27] Buus S Rockberg J Forsstr Oumlm BO Nilsson P Uhlen M Schafer-Nielsen C High-resolution mapping of linear antibody epitopes using ultrahigh-density peptide microarrays Mol Cell Proteomics 2012 11 1790 1800 10.1074/mcp.M112.020800 22984286 PMC3518105

[B28] Yohai VJ High breakdown-point and high efficiency estimates for regression The Annals of Statistics 1987 15 642 665 10.1214/aos/1176350366

[B29] Koller M Stahel WA Sharpening Wald-type inference in robust regression for small samples Computational Statistics and Data Analysis 2011 55 2504 2515 10.1016/j.csda.2011.02.014

[B30] Khalili K Sariyer IK Safak M Small tumor antigen of polyomaviruses: role in viral life cycle and cell transformation J Cell Physiol 2008 215 309 319 10.1002/jcp.21326 18022798 PMC2716072

[B31] Poletaev AB The immunological homunculus (immunculus) in normal state and pathology Biochemistry (Mosc) 2002 67 600 608 10.1023/A:1015514732179 12059783

[B32] Madi A Bransburg-Zabary S Kenett DY Ben-Jacob E Cohen IR The natural autoantibody repertoire in newborns and adults: a current overview Adv Exp Med Biol 2012 750 198 212 10.1007/978-1-4614-3461-0_15 22903676

[B33] Randhawa P Viscidi R Carter JJ Galloway DA Culp TD Huang C Ramaswami B Christensen ND Identification of species-specific and cross-reactive epitopes in human polyomavirus capsids using monoclonal antibodies J Gen Virol 2009 90 634 639 10.1099/vir.0.008391-0 19218208 PMC3075566

[B34] Wang M Tzeng TY Fung CY Ou WC Tsai RT Lin CK Tsay GJ Chang D Human anti-JC virus serum reacts with native but not denatured JC virus major capsid protein VP1 J Virol Methods 1999 78 171 176 10.1016/S0166-0934(98)00180-3 10204707

[B35] Gaseitsiwe S Valentini D Mahdavifar S Reilly M Ehrnst A Maeurer M Peptide microarray-based identification of Mycobacterium tuberculosis epitope binding to HLA-DRB1*0101, DRB1*1501, and DRB1*0401 Clin Vaccine Immunol 2010 17 168 175 10.1128/CVI.00208-09 19864486 PMC2812096

[B36] Valentini D Gaseitsiwe S Maeurer M Humoral 'reactome' profiles using peptide microarray chips Trends Immunol 2010 31 399 400 10.1016/j.it.2010.08.007 20920889

[B37] Price JV Tangsombatvisit S Xu G Yu J Levy D Baechler EC Gozani O Varma M Utz PJ Liu CL On silico peptide microarrays for high-resolution mapping of antibody epitopes and diverse protein-protein interactions Nat Med 2012 9 1434 1440 22902875 10.1038/nm.2913 PMC3491111

[B38] Andresen H Grotzinger C Zarse K Kreuzer OJ Ehrentreich-Forster E Bier FF Functional peptide microarrays for specific and sensitive antibody diagnostics Proteomics 2006 6 1376 1384 10.1002/pmic.200500343 16456884 PMC7167710

[B39] Fernandez L Chan WC Egido M Gomara MJ Haro I Synthetic peptides derived from an N-terminal domain of the E2 protein of GB virus C in the study of GBV-C/HIV-1 co-infection J Pept Sci 2013 18 326 335 22438139 10.1002/psc.2403

[B40] R-Core-Team R: A language and environment for statistical computing 2012 Vienna, Austria: R Foundation for Statistical Computing http://www.r-project.org/

[B41] Gentleman RC Carey VJ Bates DM Bolstad B Dettling M Dudoit S Ellis B Gautier L Ge Y Gentry J Bioconductor: open software development for computational biology and bioinformatics Genome Biol 2004 5 R80 10.1186/gb-2004-5-10-r80 15461798 PMC545600

[B42] Pages H Aboyoun P Gentleman R DebRoy S Biostrings: String objects representing biological sequences, and matching algorithms R package version 2241 2012

[B43] Hothorn T Hornik K van de Wiel MA Zeileis A Implementing a Class of Permutation Tests: The coin Package J Stat Software 2008 28 1 23

[B44] Rousseeuw P Croux C Todorov V Ruckstuhl A Salibian-Barrera M Verbeke T Koller M Maechler M robustbase: Basic Robust Statistics. R package version 0.8-0 2011 http://CRAN.R-project.org/package=robustbase

