# Supplementary material for: An antibody response to human polyomavirus 15-mer peptides is highly abundant in healthy human subjects
Source: Virol J. 2013 Jun 12;10:192. doi: 10.1186/1743-422X-10-192 (PMC3691923; doi:10.1186/1743-422X-10-192)
Supplement: Additional file 1 — Unique polyomavirus pentapeptides sequences. List of polyomavirus-derived pentapeptides with no homology to the human proteome. [file 1743-422X-10-192-S1.doc]

Supplement Figure 1: List of all HPyV penta-peptides with no match in the human genome. Each penta-peptide is preceded by its codon position in the viral protein. The analysis was done using the polyoma virus sequences provided with the RefSeq database (ftp://ftp.ncbi.nlm.nih.gov/refseq/release/viral/), except for MCV LTAg which was replaced by AEM01097. The human proteome used in the analysis is available at <http://www.uniprot.org/faq/48>).

JCV Agno: 18-WSGTK, 38-DFCTG, 40-CTGED,

JCV LTAg: 26-NIPVM, 59-YKKME, 61-KMEQG, 83-PTYGT, 89-EWESW, 90-WESWW, 91-ESWWN, 92-SWWNT, 93-WWNTF, 97-FNEKW, 108-HEEMF, 209-INNYC, 210-NNYCQ, 212-YCQKL, 217-CTFSF, 255-EHDFN, 256-HDFNP, 268-QVSWK, 281-TKCED, 289-LMGMY, 291-GMYLD, 292-MYLDF, 300-PQQCK, 309-KDQPN, 313-NHFNH, 314-HFNHH, 317-HHEKH, 320-KHYYN, 324-NAQIF, 337-ICQQA, 338-CQQAV, 352-DSIHM, 353-SIHMT, 354-IHMTR, 355-HMTRE, 376-IFGAH, 385-LEQYM, 386-EQYMA, 392-VAWIH, 393-AWIHC, 400-PQMDT, 466-IDQFM, 467-DQFMV, 468-QFMVV, 470-MVVFE, 490-HGISN, 518-RTQVF, 526-IVTMN, 580-LIWFR, 597-IVQWK, 609-SMYTF, 616-MKANV, 649-QSQCF, 669-TFHIC, 675-GFQCF,

JCV stAg: 26-NIPVM, 59-YKKME, 61-KMEQG, 85-DFPPN, 91-DTLYC, 92-TLYCK, 95-CKEWP, 96-KEWPN, 97-EWPNC, 98-WPNCA, 99-PNCAT, 100-NCATN, 101-CATNP, 107-VHCPC, 110-PCLMC, 111-CLMCM, 131-LVWID, 133-WIDCY, 134-IDCYC, 135-DCYCF, 137-YCFDC, 138-CFDCF, 144-QWFGC, 164-DTPYR,

JCV VP1: 39-EVECF, 40-VECFL, 45-TPEMG, 73-PNRDM, 74-NRDML, 77-MLPCY, 79-PCYSV, 96-TCGNI, 98-GNILM, 102-MWEAV, 120-NVHSN, 128-THDNG, 129-HDNGA, 158-LFNYR, 160-NYRTK, 182-MNTEH, 185-EHKAY, 197-PVECW, 198-VECWV, 199-ECWVP, 200-CWVPD, 261-GMFTN, 262-MFTNR, 292-YPISF, 314-MYGMD, 336-DPDMM, 338-DMMRY, 339-MMRYV, 340-MRYVD,

JCV VP2 64-TPETY, 98-GYRFF, 99-YRFFA, 101-FFADW, 102-FADWD, 103-ADWDH, 104-DWDHK, 105-WDHKV, 140-AFVNN, 141-FVNNI, 143-NNIHY, 149-DPRHW, 150-PRHWG, 162-SQAFW, 163-QAFWN, 164-AFWNL, 199-ETTWA, 209-ANLYN, 215-ISDYY, 245-HSYTQ, 291-APQWM, 292-PQWML, 293-QWMLP.

BKV Agno: 16-KTWTG, 38-EFCRG,

BKV LTAg: 20-ERAAW, 22-AAWGN, 59-YKKME, 74-FGTWS, 83-PTYGT, 86-GTEEW, 89-EWESW, 90-WESWW, 93-WWSSF, 97-FNEKW, 108-HEDMF, 186-ISRHM, 187-SRHMC, 188-RHMCA, 189-HMCAG, 190-MCAGH, 194-HNIIF, 211-NNFCQ, 212-NFCQK, 218-CTFSF, 269-QVSWK, 282-TKCED, 293-MYLEF, 296-EFQYN, 297-FQYNV, 298-QYNVE, 312-QPYHF, 316-FKYHE, 318-YHEKH, 319-HEKHF, 338-ICQQA, 339-CQQAV, 350-KRVDT, 353-DTLHM, 355-LHMTR, 356-HMTRE, 366-RFNHI, 377-IFGAH, 386-LEQYM, 387-EQYMA, 393-VAWLH, 395-WLHCL, 402-KMDSV, 412-HCIVF, 413-CIVFN, 420-KRRYW, 421-RRYWL, 445-CGGKA, 468-DQYMV, 470-YMVVF, 471-MVVFE, 490-GHGIN, 550-RPKIY, 581-LIWFR, 589-DFATD, 599-VEWKE, 610-SMYTF, 618-KYNIC, 619-YNICM, 620-NICMG, 621-ICMGK, 622-CMGKC, 623-MGKCI, 650-QSQCS, 682-GFQCF, 684-QCFKR,

BKV stag: 20-ERAAW, 22-AAWGN, 59-YKKME, 74-FGTWS, 82-VCADF, 83-CADFP, 85-DFPLC, 91-DTLYC, 92-TLYCK, 95-CKEWP, 97-EWPIC, 98-WPICS, 107-VHCPC, 108-HCPCM, 109-CPCML, 131-LVWID, 133-WIDCY, 134-IDCYC, 135-DCYCI, 136-CYCID, 137-YCIDC, 140-DCFTQ, 142-FTQWF, 144-QWFGL, 153-ETLQW, 154-TLQWW, 155-LQWWV, 156-QWWVQ, 157-WWVQI, 158-WVQII, 165-TPFRD,

BKV VP1: 6-RKGEC, 8-GECPG, 47-EVECF, 48-VECFL, 84-KMLPC, 85-MLPCY, 87-PCYST, 110-MWEAV, 135-KVHEH, 136-VHEHG, 137-HEHGG, 147-GSNFH, 149-NFHFF, 150-FHFFA, 166-LMNYR, 167-MNYRS, 190-MNTDH, 191-NTDHK, 202-NAYPV, 205-PVECW, 206-VECWV, 207-ECWVP, 208-CWVPD, 217-ENARY, 236-HVTNT, 277-TQQWR, 284-ARYFK, 285-RYFKI, 300-YPISF,

BKV VP2: 95-AQVGY, 96-QVGYK, 97-VGYKF, 103-DDWDH, 104-DWDHK, 105-WDHKV, 113-GLYQQ, 116-QQSGM, 141-FVNNI, 143-NNIQY, 149-DPRHW, 150-PRHWG, 165-LWHVI, 202-WTIVN, 208-PINFY, 211-FYNYI, 212-YNYIQ, 215-IQQYY, 217-QYYSD, 241-VHFGH, 242-HFGHT, 243-FGHTY, 245-HTYSI, 259-TQRMD, 261-RMDLR, 290-APQWM, 291-PQWML, 292-QWMLP.

SV40 Agno:15-RRSWT, 18-WTESK, 38-QFCEG, 39-FCEGE,

SV40 LTAg: 59-YKKME, 66-VKYAH, 75-GGFWD, 77-FWDAT, 84-PTYGT, 90-EWEQW, 91-WEQWW, 94-WWNAF, 95-WNAFN, 189-SYNHN, 190-YNHNI, 208-INNYA, 209-NNYAQ, 216-CTFSF, 254-EHDFN, 255-HDFNP, 267-QVSWK, 274-TEYAM, 280-TKCDD, 291-MYLEF, 299-FEMCL, 311-PSHYK, 312-SHYKY, 314-YKYHE, 316-YHEKH, 320-HYANA, 336-ICQQA, 337-CQQAV, 369-LDRMD, 371-RMDIM, 372-MDIMF, 373-DIMFG, 383-DIEEW, 384-IEEWM, 387-WMAGV, 391-VAWLH, 393-WLHCL, 400-KMDSV, 410-KCMVY, 411-CMVYN, 443-CGGKA, 464-AIDQF, 525-IVTMN, 577-LMLIW, 578-MLIWY, 580-IWYRP, 581-WYRPV, 597-VEWKE, 611-VYQKM, 613-QKMKF, 614-KMKFN, 617-FNVAM, 678-QSVHD, 685-QPYHI, 687-YHICR, 690-CRGFT, 691-RGFTC,

SV40 stAg: 59-YKKME, 66-VKYAH, 75-GGFWD, 77-FWDAT, 91-GVDAM, 93-DAMYC, 94-AMYCK, 95-MYCKQ, 97-CKQWP, 98-KQWPE, 99-QWPEC, 100-WPECA, 107-MSANC, 108-SANCI, 109-ANCIC, 110-NCICL, 121-KHENR, 134-VWVDC, 137-DCYCF, 139-YCFDC, 140-CFDCF, 142-DCFRM, 143-CFRMW, 144-FRMWF, 145-RMWFG, 158-LLWCD, 160-WCDII, 162-DIIGQ, 163-IIGQT,

SV40 VP1: 49-EVECF, 50-VECFL, 56-PQMGN, 89-PCYSV, 106-TCGNI, 108-GNILM, 112-MWEAV, 132-HSGTQ, 149-GSNFH, 151-NFHFF, 152-FHFFA, 169-ANYRT, 170-NYRTK, 190-QQMNT, 191-QMNTD, 192-MNTDH, 193-NTDHK, 202-KDNAY, 204-NAYPV, 207-PVECW, 208-VECWV, 209-ECWVP, 210-CWVPD, 223-YFGTY, 267-VDICG, 287-RYFKI, 302-YPISF, 322-QPMIG, 323-PMIGM, 335-RVYED, 360-TTRMQ,

SV40 VP2: 94-AQVGY, 97-GYRFF, 98-YRFFS, 101-FSDWD, 102-SDWDH, 103-DWDHK, 104-WDHKV, 112-GLYQQ, 124-YRPDD, 138-QTFVH, 148-DPRHW, 149-PRHWG, 151-HWGPT, 152-WGPTL, 161-SQAFW, 162-QAFWR, 163-AFWRV, 164-FWRVI, 165-WRVIQ, 206-APVNW, 207-PVNWY, 208-VNWYN, 209-NWYNS, 210-WYNSL, 225-RPTMV, 241-SFGHT, 242-FGHTY, 244-HTYDN, 246-YDNID, 259-TERWE, 291-APQWM, 292-PQWML, 293-QWMLP.

KIV LTAg: 20-DMSCW, 21-MSCWG, 23-CWGNL, 30-MRRQY, 38-CKEYH, 77-DNIWQ, 79-IWQSS, 86-PTYGT, 90-TPDWD, 91-PDWDE, 92-DWDEW, 93-WDEWW, 94-DEWWS, 95-EWWSQ, 96-WWSQF, 100-FNTYW, 101-NTYWE, 161-SNKCI, 162-NKCIT, 163-KCITC, 169-VVHTT, 171-HTTRE, 186-QKYQC, 195-KHAFY, 196-HAFYN, 207-LTPHK, 208-TPHKH, 210-HKHRV, 218-NNFCK, 219-NFCKG, 220-FCKGH, 221-CKGHC, 229-FLFCK, 231-FCKGV, 242-YSRMC, 244-RMCRQ, 245-MCRQP, 247-RQPFN, 249-PFNLC, 282-WNQIA, 304-YIRFA, 323-ATHKR, 328-VHVQN, 332-NHENA, 347-NACTQ, 361-RYNCL, 381-FNEMD, 397-YMASI, 399-ASIAW, 400-SIAWY, 404-YTGLN, 427-PKHRY, 428-KHRYW, 453-CGGKA, 475-LDQYM, 476-DQYMV, 478-YMVVF, 479-MVVFE, 487-GQIGI, 498-GNGVN, 499-NGVNN, 535-IVTMN, 538-MNEYC, 539-NEYCI, 590-IWYRP, 591-WYRPV, 606-VVYWK, 607-VYWKE, 614-DNYIG, 622-FATMQ, 624-TMQMN,

KIV stAg: 20-DMSCW, 21-MSCWG, 23-CWGNL, 30-MRRQY, 38-CKEYH, 77-DNIWQ, 79-IWQSS, 84-QVYCK, 87-CKDLC, 89-DLCCN, 101-IYGDY, 105-YYEAY, 106-YEAYI, 107-EAYIM, 110-IMKQW, 111-MKQWD, 113-QWDVC, 114-WDVCI, 117-CIHGY, 118-IHGYN, 120-GYNHE, 121-YNHEC, 122-NHECQ, 123-HECQC, 125-CQCIH, 127-CIHCI, 133-SKYHK, 148-VWIEC, 149-WIECY, 151-ECYCY, 152-CYCYK, 153-YCYKC, 154-CYKCY, 157-CYREW, 158-YREWF, 160-EWFFF, 161-WFFFP, 165-PISMQ, 167-SMQTF, 170-TFFFW, 172-FFWKV, 173-FWKVI, 174-WKVII,

KIV VP1: 52-LFVKP, 70-PTPHY, 72-PHYWS, 73-HYWSI, 100-TTVCY, 119-SECDM, 121-CDMKV, 122-DMKVW, 126-WELYR, 128-LYRME, 146-NTNGV, 200-YNSNT, 215-SVESW, 217-ESWAP, 218-SWAPD, 219-WAPDP, 224-SRNDN, 225-RNDNC, 226-NDNCR, 229-CRYFG, 231-YFGRV, 272-ITCAD, 274-CADML, 289-MARFF, 306-FTMNV, 308-MNVLY, 314-QVFNR,

KIV VP2: 134-HKPIH, 136-PIHAP, 137-IHAPY, 141-YSGMA, 162-PDWLF, 163-DWLFN, 187-AFGIW, 281-AAYNF, 295-DGFNA, 307-LGQWI, 309-QWISF, 322-HYATP, 323-YATPD, 339-DTYKI.

WUV LTAg:20-DMTCW, 22-TCWGN, 23-CWGNL, 86-PTYGT, 90-TPDWD, 91-PDWDY, 92-DWDYW, 93-WDYWW, 95-YWWSQ, 96-WWSQF, 101-NSYWE, 102-SYWEE, 134-QCSQA, 175-AFVIH, 195-KFKCN, 196-FKCNF, 203-RHSYY, 226-NNFCK, 227-NFCKG, 228-FCKGY, 229-CKGYC, 231-GYCTI, 232-YCTIS, 233-CTISF, 237-FLFCK, 239-FCKGV, 250-YSRMT, 277-EDLYG, 285-DQLNW, 335-LHMEH, 336-HMEHQ, 352-QKNVC, 354-NVCQQ, 407-GVAWY, 436-KRYWV, 460-CGGKA, 481-AIDQF, 542-IVTMN, 564-FTPKR, 587-QSGMC, 594-IMLIW, 595-MLIWC, 596-LIWCR, 597-IWCRP, 598-WCRPV, 604-DFHPC, 612-KVVYW, 613-VVYWK, 614-VYWKE, 630-ADMQM, 631-DMQMN,

WUV stAg:20-DMTCW, 22-TCWGN, 23-CWGNL, 87-CTELC, 90-LCCNF, 116-KDWDI, 117-DWDIC, 118-WDICL, 126-YYLCN, 127-YLCNC, 128-LCNCF, 130-NCFYC, 131-CFYCF, 143-KYKIF, 149-KPPMW, 150-PPMWI, 152-MWIEC, 153-WIECY, 155-ECYCY, 156-CYCYR, 158-CYRCY, 161-CYREW, 162-YREWF, 163-REWFG, 165-WFGFE, 172-AETFF, 175-FFYWK,

WUV VP1: 6-KPACT, 11-AKPGR, 28-PKQVR, 54-LFVKP, 65-GTTPH, 68-PHYWS, 69-HYWSI, 91-TTVCY, 110-SECDM, 113-DMLIW, 114-MLIWE, 116-IWELY, 117-WELYR, 119-LYRME, 167-PTEKY, 173-GPAQY, 176-QYTVN, 177-YTVNP, 209-ESWVA, 211-WVADP, 216-SRNDN, 217-RNDNC, 218-NDNCR, 221-CRYFG, 223-YFGRM, 264-ITCAD, 272-VNKNR, 307-VFNKP,

WUV VP2: 162-PDWVF, 164-WVFNF, 165-VFNFI, 187-AYGIW, 188-YGIWT, 191-WTSYY, 235-NAIVE, 293-TYNFV, 294-YNFVY, 306-DGFNA, 318-LGQWI, 321-WISMP, 324-MPGAT, 335-AAPDW, 336-APDWI.

MCV LTAg: 20-APNCY, 49-PVIMM, 56-NTLWS, 59-WSKFQ, 62-FQQNI, 86-GTTKF, 89-KFKEW, 90-FKEWW, 91-KEWWR, 92-EWWRS, 93-WWRSG, 104-AYEYG, 106-EYGPN, 110-NPHGT, 159-RGPDI, 160-GPDIP, 213-FCDES, 322-YLSHA, 334-SCFAI, 357-VDFKS, 359-FKSRH, 360-KSRHA, 362-RHACE, 365-CELGC, 386-NFCST, 390-TFCTI, 392-CTISF, 403-NKMPE, 405-MPEMY, 406-PEMYN, 409-YNNLC, 414-KPPYK, 427-LNYEF, 428-NYEFQ, 438-ASCNW, 439-SCNWN, 447-EFACE, 449-ACEYE, 451-EYELD, 452-YELDD, 455-DDHFI, 456-DHFII, 470-PFPCQ, 471-FPCQK, 475-KCENR, 485-HKAHE, 487-AHEAH, 488-HEAHH, 491-HHSNA, 507-ICQQA, 524-LEMTR, 553-YYMGG, 554-YMGGV, 557-GVAWY, 558-VAWYC, 559-AWYCC, 560-WYCCL, 561-YCCLF, 562-CCLFE, 588-NIWFK, 589-IWFKG, 590-WFKGP, 616-NINCP, 617-INCPS, 633-DKFMV, 636-MVVFE, 642-VKGQN, 684-KHQIF, 685-HQIFP, 688-FPPCI, 689-PPCIV, 694-TANDY, 745-CLIWC, 747-IWCLP, 754-TFKPC, 755-FKPCL, 762-EIKNW, 764-KNWKQ, 765-NWKQI, 778-KFCQM, 780-CQMIE,

MCV stAg: 20-APNCY, 49-PVIMM, 56-NTLWS, 59-WSKFQ, 62-FQQNI, 81-TKFPW, 92-KDYMQ, 96-QSGYN, 100-NARFC, 107-PGCML, 109-CMLKQ, 116-DSKCA, 118-KCACI, 119-CACIS, 120-ACISC, 137-KQKNC, 141-CLTWG, 142-LTWGE, 143-TWGEC, 144-WGECF, 146-ECFCY, 147-CFCYQ, 149-CYQCF, 152-CFILW, 154-ILWFG, 156-WFGFP, 165-SFDWW, 166-FDWWQ, 167-DWWQK, 168-WWQKT,

MCV VP1: 23-GCCPN, 25-CPNVA, 62-RMGVN, 72-TTSNW, 73-TSNWY, 75-NWYTY, 77-YTYTY, 81-YDLQP, 114-DITCD, 115-ITCDT, 121-QMWEA, 122-MWEAI, 138-LINVH, 140-NVHYW, 141-VHYWD, 142-HYWDM, 143-YWDMK, 144-WDMKR, 146-MKRVH, 149-VHDYG, 160-GVNYH, 162-NYHMF, 163-YHMFA, 164-HMFAI, 183-QTEYP, 185-EYPKT, 205-MTPKN, 223-DGNYP, 224-GNYPI, 225-NYPIE, 226-YPIEV, 228-IEVWC, 230-VWCPD, 231-WCPDP, 241-SRYYG, 277-CKGDG, 307-RYFNV, 317-WVKNP, 334-NLMPK, 348-DNQVE, 355-RIYEG, 365-GNPDI, 375-KFGQE, 394-TFQSN,

MCV VP2: 66-QFSNF, 114-DVSWV, 117-WVGSN, 170-ILNSR, 174-RWVFQ, 175-WVFQT.

MWV LTAg:20-NVAAW, 22-AAWGN, 28-PLMQY, 29-LMQYK, 31-QYKYR, 32-YKYRQ, 33-KYRQA, 34-YRQAC, 76-YFPAK, 83-PTYGT, 90-WDQWW, 92-QWWEE, 98-NRGWD, 185-LYTTR, 206-MFYSL, 231-AIKNY, 254-YECYY, 255-ECYYA, 258-YALCK, 261-CKTPF, 264-PFKLI, 279-TDFCE, 280-DFCEE, 289-VVNWQ, 291-NWQQI, 292-WQQIC, 302-VQCED, 303-QCEDP, 333-KHHYK, 334-HHYKF, 335-HYKFH, 350-KDCKN, 351-DCKNQ, 358-ICQQA, 361-QATDW, 362-ATDWV, 365-WVTAQ, 387-FKYMF, 389-YMFEK, 390-MFEKM, 397-ICGEV, 401-VEICQ, 402-EICQY, 404-CQYMA, 417-MPHFD, 419-HFDEI, 478-FEIGC, 481-GCAID, 486-EYMVV, 487-YMVVF, 488-MVVFE, 494-VKGQN, 508-GMGMC, 509-MGMCN, 512-CNLDN, 547-TMNDY, 548-MNDYF, 558-QARMI, 599-LCWWQ, 600-CWWQP, 605-VIAFH, 612-IHDNV, 615-NVRYW, 616-VRYWK, 619-WKETI, 625-KYVPF, 627-VPFGM, 629-FGMYH, 631-MYHDI,

MWV stAg:20-NVAAW, 22-AAWGN, 28-PLMQY, 29-LMQYK, 31-QYKYR, 32-YKYRQ, 33-KYRQA, 34-YRQAC, 76-YFPAK, 80-KVGYF, 103-IIYIW, 105-YIWPL, 107-WPLCA, 118-KCGCV, 130-HRNDK, 137-KQKQC, 141-CLVWG, 144-WGECF, 146-ECFCY, 147-CFCYS, 149-CYSCF, 150-YSCFL, 156-WFGQE, 157-FGQEF, 160-EFGYT, 164-TSFFW, 165-SFFWW, 166-FFWWK, 167-FWWKH, 168-WWKHI, 169-WKHIM, 170-KHIMH, 172-IMHNI, 173-MHNIE, 175-NIEFD, 187-LILWV,

MWV VP1: 35-RMSCN, 40-KCPCI, 70-PDTTM, 83-RMGND, 84-MGNDV, 91-NKWYG, 92-KWYGY, 93-WYGYS, 104-NTPTI, 126-EDMTC, 127-DMTCE, 133-LYMWE, 134-YMWEA, 135-MWEAV, 152-ITLHT, 175-FHFFA, 186-DLQYC, 196-IVYPD, 228-YYPVE, 230-PVEAW, 244-SRYYG, 280-CKGDG, 290-VDICG, 293-CGVFQ, 294-GVFQM, 311-YFQVQ, 386-VTDMP,

MWV VP2: 115-QWRPD, 116-WRPDY, 117-RPDYF, 121-FDVFI, 128-YRHFE, 129-RHFEY, 130-HFEYY, 131-FEYYF, 150-SRAFW, 175-NVYNV, 178-NVGEQ, 207-NVYHN, 211-NLEMY, 214-MYYAQ, 237-ARNYG, 277-TPDWM, 278-PDWML, 279-DWMLQ.

TSV LTAg: 22-HCYGN, 23-CYGNF, 24-YGNFA, 37-MSLKY, 52-MSRLN, 57-QLWQK, 66-IYNAR, 67-YNARQ, 72-EFPTS, 79-SQHDV, 98-SWASW, 99-WASWW, 100-ASWWE, 102-WWESF, 106-FNQEW, 107-NQEWD, 115-DTMQD, 123-FCHES, 162-DSPGC, 186-VPNDF, 189-DFPDM, 212-LIYTT, 213-IYTTN, 214-YTTNE, 231-NPEFK, 234-FKSRH, 236-SRHSF, 262-LCVTH, 280-QVECY, 283-CYRCM, 284-YRCMC, 285-RCMCS, 286-CMCSE, 287-MCSEP, 288-CSEPF, 315-VVNWN, 335-LIMAH, 350-CSKCT, 359-KAHYN, 360-AHYNY, 361-HYNYH, 368-HHKNA, 375-FKECK, 376-KECKT, 412-RFKLM, 435-GVAWY, 438-WYSCL, 463-KKRNC, 496-NCPAD, 507-GCAID, 509-AIDQF, 512-QFVVI, 569-CLVTM, 585-RFAYV, 592-FTPKH, 625-VWYCP, 626-WYCPI, 627-YCPIT, 628-CPITM, 630-ITMFS, 639-EDVKY, 642-KYWKD, 648-LCKYM, 649-CKYMG, 650-KYMGH, 654-HTNFA,

TSV stAg: 22-HCYGN, 23-CYGNF, 24-YGNFA, 37-MSLKY, 52-MSRLN, 57-QLWQK, 66-IYNAR, 67-YNARQ, 72-EFPTS, 82-GSWYW, 84-WYWEA, 85-YWEAN, 86-WEANL, 107-IKHWP, 108-KHWPQ, 109-HWPQC, 110-WPQCA, 122-KCLTC, 123-CLTCK, 126-CKIGL, 133-HVYKQ, 134-VYKQM, 135-YKQMH, 140-QKKCV, 146-WGECF, 148-ECFCY, 151-CYKCY, 154-CYCAW, 155-YCAWF, 156-CAWFG, 157-AWFGE, 168-SLWAW, 170-WAWSC, 171-AWSCI, 194-FNWGK,

TSV VP1: 6-KGEGC, 10-CARKC, 55-TRMGQ, 56-RMGQN, 67-YGYSE, 113-MWEAV, 130-VNVHM, 139-MYDDK, 150-EGMNF, 151-GMNFH, 152-MNFHM, 154-FHMFA, 187-PIKHQ, 193-TQGLN, 195-GLNPH, 198-PHYKQ, 211-PVECW, 212-VECWC, 214-CWCPD, 215-WCPDP, 225-TRYYG, 261-CKGDG, 267-YVSCC, 269-SCCDI, 281-DGDMQ, 283-DMQYR, 284-MQYRG, 317-TGLMP, 326-QPMDN, 327-PMDNG, 354-MVRYI, 357-YIDKF,

TSV VP2: 43-MGLMT, 44-GLMTV, 67-FSLMH, 70-MHALP, 100-GIRYG, 101-IRYGY, 124-WRPWD, 125-RPWDY, 126-PWDYY, 127-WDYYD, 137-VQTFA, 141-AHYLN, 148-DHWAS, 159-SRYVW, 161-YVWDA, 168-HEGRH, 172-HQIGH, 182-MIRGT, 185-GTNHF, 270-RVCPD, 271-VCPDW, 272-CPDWM, 273-PDWML, 274-DWMLP, 290-TFGYY, 291-FGYYL, 292-GYYLR.

HPyV6 LTAg:20-SMACW, 21-MACWG, 22-ACWGN, 23-CWGNL, 37-ACKKY, 73-SPTWC, 74-PTWCF, 75-TWCFS, 92-GWEQW, 93-WEQWW, 94-EQWWA, 95-QWWAD, 96-WWADF, 97-WADFN, 101-NRGWD, 160-YLSHA, 170-CYTCF, 183-WETLY, 203-KCNDN, 205-NDNTG, 212-LYCIT, 214-CITPR, 223-SAMLN, 230-SKCCT, 231-KCCTI, 233-CTISF, 245-KSAEC, 249-CYMAL, 254-QGDEF, 270-HSYDF, 271-SYDFQ, 281-EECDW, 282-ECDWN, 283-CDWNQ, 284-DWNQV, 285-WNQVA, 306-YYIEF, 309-EFAND, 316-SCMKC, 318-MKCKK, 324-VKVHK, 326-VHKHH, 327-HKHHE, 329-HHEVH, 332-VHFHN, 353-ACDRV, 388-QYAGG, 461-VNCTP, 462-NCTPD, 463-CTPDK, 473-GCAID, 474-CAIDK, 477-DKFMC, 478-KFMCV, 479-FMCVI, 500-CGMTN, 502-MTNLD, 516-VPVNM, 542-YIIPC, 545-PCTVK, 546-CTVKA, 560-HKPCL, 588-ALIWW, 589-LIWWE, 592-WEPVE, 606-VVNWK, 608-NWKQT, 609-WKQTF, 613-FERWV, 615-RWVSF, 617-VSFGM, 619-FGMYQ,

HPyV6 stAg:20-SMACW, 21-MACWG, 22-ACWGN, 23-CWGNL, 37-ACKKY, 73-SPTWC, 74-PTWCF, 75-TWCFS, 84-DDWGI, 85-DWGIP, 105-VWDFR, 106-WDFRL, 119-CKCLH, 130-HKKQV, 142-TIWGK, 143-IWGKC, 144-WGKCW, 146-KCWCY, 147-CWCYK, 148-WCYKC, 149-CYKCY, 152-CYCLW, 153-YCLWF, 154-CLWFG, 156-WFGLP, 163-ADSFM, 164-DSFMW, 165-SFMWW, 166-FMWWT, 167-MWWTH, 168-WWTHI, 169-WTHII, 172-IIYQS,

HPyV6 VP1: 1-MPCHR, 2-PCHRK, 3-CHRKG, 60-GAPYT, 74-SLCYT, 90-ALCDD, 109-IFTPQ, 119-YIRAQ, 131-GSQMY, 134-MYFWA, 135-YFWAC, 136-FWACG, 137-WACGG, 154-ERMNV, 186-ANFPI, 189-PIEIW, 190-IEIWS, 203-CRYFG, 255-VGYAG, 290-VKHPY, 296-VDMMF, 297-DMMFR, 313-GTQPN, 314-TQPNA, 323-VMEQM,

HPyV6 VP2: 97-NYNPG, 119-NTTMA, 127-WLPQV, 163-VRGIW, 164-RGIWT, 167-WTSYY, 220-NYAVN, 226-NRQWE, 227-RQWET, 248-NYDMQ, 249-YDMQN, 266-FHDEG, 281-NTGQY, 283-GQYCI, 286-CIPQW, 287-IPQWL, 309-QKRKW, 312-KWTNS.

HPyV7 LTAg: 20-NMACW, 21-MACWG, 22-ACWGN, 23-CWGNL, 74-PMWHY, 76-WHYSS, 93-AWDQW, 94-WDQWW, 95-DQWWQ, 96-QWWQD, 97-WWQDF, 98-WQDFN, 100-DFNKG, 101-FNKGW, 108-DLYCT, 109-LYCTE, 157-DFPEC, 175-CYTCF, 177-TCFLC, 179-FLCYT, 185-YEKSM, 188-SMLLY, 205-GAYNC, 207-YNCVD, 208-NCVDG, 234-CKKHC, 235-KKHCT, 253-ECYKA, 275-HSYDF, 276-SYDFQ, 286-DDCDW, 287-DCDWN, 288-CDWNF, 289-DWNFV, 296-FAADM, 307-LIMGY, 309-MGYYM, 311-YYMEF, 312-YMEFA, 331-AHQHH, 334-HHEKH, 338-HWANA, 339-WANAK, 351-QKGIA, 367-RVLMM, 368-VLMME, 377-DLMVM, 386-QFKVL, 402-QLIGA, 416-QFTIK, 478-GCAID, 479-CAIDK, 483-KYMVV, 511-DNMRD, 512-NMRDY, 526-ERKHI, 537-FPPCI, 542-ITCNE, 544-CNEYA, 594-ALIWW, 595-LIWWE, 612-VVNWK, 614-NWKQT, 615-WKQTF, 622-WVSYG, 626-GMFQT, 627-MFQTM, 640-DPFEG, 646-LINDP, 666-IGSMN,

HPyV7 stAg: 20-NMACW, 21-MACWG, 22-ACWGN, 23-CWGNL, 74-PMWHY, 76-WHYSS, 82-EVSFW, 84-SFWDI, 105-VWNYN, 106-WNYNL, 107-NYNLC, 117-RACCC, 118-ACCCI, 119-CCCIH, 120-CCIHC, 121-CIHCI, 137-YAKDH, 147-WGKCW, 150-CWCFD, 151-WCFDC, 152-CFDCY, 156-YLDWF, 157-LDWFG, 167-ESFMW, 168-SFMWW, 169-FMWWS, 170-MWWSH, 171-WWSHI, 172-WSHII, 174-HIIFQ,

HPyV7 VP1: 8-NGPTQ, 34-ETQYK, 35-TQYKV, 54-GNFQS, 61-LPYPM, 63-YPMSD, 75-LCYSV, 91-MCEDT, 92-CEDTM, 93-EDTMI, 95-TMIVW, 96-MIVWE, 97-IVWEA, 110-FAPQM, 119-YQRAN, 135-YFWAC, 136-FWACG, 137-WACGG, 181-NAANF, 189-LWVAD, 190-WVADP, 197-NDNTR, 202-YFGRV, 218-YGNQS, 247-DMVGM, 264-YSNQR, 266-NQRTV, 274-YGRFF, 280-VHCRQ, 287-IKHPY, 293-VDMMF, 294-DMMFR, 317-QEVTM, 339-FAPTS,

HPyV7 VP2: 129-QAWPW, 130-AWPWG, 131-WPWGG, 159-YNIAR, 163-RGIWT, 166-WTSYY, 193-RQNII, 204-IEMAP, 246-VMYDT, 247-MYDTQ, 265-FHDEG, 266-HDEGT, 271-WVSFQ, 274-FQGEE, 281-TPQYT, 284-YTIPQ, 285-TIPQW, 286-IPQWM, 287-PQWML, 288-QWMLF, 304-ENKHA, 313-RKWTH.

HPyV9 LTAg:22-AAWGN, 69-IRSNC, 94-SYCER, 95-YCERK, 107-SWGKW, 110-KWWRE, 111-WWREF, 112-WREFV, 121-DDLFC, 184-DFPMC, 185-FPMCL, 186-PMCLY, 187-MCLYS, 194-SHAIY, 201-KTMNC, 202-TMNCF, 205-CFLIY, 207-LIYTT, 237-YKDDG, 262-HFCVA, 268-CTFSF, 303-VSMFD, 304-SMFDF, 318-WQEIC, 359-KFHYN, 361-HYNYH, 363-NYHSK, 364-YHSKH, 369-HANAC, 384-ICQQA, 385-CQQAV, 401-VECTR, 402-ECTRM, 403-CTRME, 424-LHGEI, 432-RWMSG, 436-GVAWY, 437-VAWYT, 439-WYTIL, 444-LDNSW, 447-SWDVF, 482-ASAFM, 483-SAFMH, 484-AFMHF, 486-MHFFD, 495-NINCP, 496-INCPA, 508-GCAID, 510-AIDQF, 511-IDQFC, 571-IMTMN, 572-MTMNE, 590-HIHFH, 591-IHFHC, 592-HFHCK, 593-FHCKT, 594-HCKTY, 595-CKTYL, 654-ITQFG, 668-KDPLH, 672-HGIVI,

HPyV9 stAg:22-AAWGN, 69-IRSNC, 80-VAWYF, 81-AWYFW, 82-WYFWD, 85-WDENF, 108-YPDCI, 111-CITYN, 118-SCCCI, 120-CCIVC, 128-QQHKS, 129-QHKST, 138-KKPCL, 139-KPCLV, 140-PCLVW, 141-CLVWG, 144-WGECF, 146-ECFCY, 149-CYKCY, 150-YKCYL, 152-CYLLW, 156-WFGFP, 165-SFNYW, 166-FNYWT, 173-MRNMD,

HPyV9 VP1:48-QIEAY, 56-RMGNN, 57-MGNNN, 60-NNPTD, 97-IKLPM, 104-EDMTC, 105-DMTCD, 106-MTCDT, 110-TLLMW, 113-MWEAV, 153-TLHMF, 177-YPTDM, 205-DGKYP, 209-PVEVW, 223-TRYYG, 238-VMQFT, 274-IHTNY, 283-NWRGL, 289-RYFNV, 357-KFCQN,

HPyV9 VP2:89-GIFFQ, 90-IFFQT, 107-TTFGY, 123-TPWFP, 124-PWFPQ, 125-WFPQV, 148-DWGES, 158-GREIW, 160-EIWRN, 161-IWRNI, 162-WRNIM, 164-NIMRQ, 172-QIGYT, 186-NEFQH, 187-EFQHM, 190-HMLAQ, 197-ENARW, 198-NARWA, 227-IQLRQ, 267-HYVQH, 283-SQDWM, 285-DWMLP, 298-ITPTW, 314-YGPPK, 325-SMSCK, 341-TPCQR, 342-PCQRR.
